# The Role of Imaging in Bladder Cancer Diagnosis and Staging

**DOI:** 10.3390/diagnostics10090703

**Published:** 2020-09-16

**Authors:** Samuel J. Galgano, Kristin K. Porter, Constantine Burgan, Soroush Rais-Bahrami

**Affiliations:** 1 Department of Radiology, University of Alabama at Birmingham, Birmingham, AL 35249, USA; samuelgalgano@uabmc.edu (S.J.G.); kkporter@uabmc.edu (K.K.P.); cburgan@uabmc.edu (C.B.); 2 O’Neal Comprehensive Cancer Center at UAB, University of Alabama at Birmingham, Birmingham, AL 35249, USA; 3 Department of Urology, University of Alabama at Birmingham, Birmingham, AL 35249, USA

**Keywords:** urothelial carcinoma, magnetic resonance imaging, positron emission tomography, computed tomography, cancer staging, muscle-invasive bladder cancer

## Abstract

Bladder cancer (BC) is the most common cancer of the urinary tract in the United States. Imaging plays a significant role in the management of patients with BC, including the locoregional staging and evaluation for distant metastatic disease, which cannot be assessed at the time of cystoscopy and biopsy/resection. We aim to review the current role of cross-sectional and molecular imaging modalities for the staging and restaging of BC and the potential advantages and limitations of each imaging modality. CT is the most widely available and frequently utilized imaging modality for BC and demonstrates good performance for the detection of nodal and visceral metastatic disease. MRI offers potential value for the locoregional staging and evaluation of muscular invasion of BC, which is critically important for prognostication and treatment decision-making. FDG-PET/MRI is a novel hybrid imaging modality combining the advantages of both MRI and FDG-PET/CT in a single-setting comprehensive staging examination and may represent the future of BC imaging evaluation.

## 1. Introduction

Bladder cancer (BC) is the most common cancer of the urinary tract in the United States, accounting for 80,470 new cases and 17,670 deaths in 2019 [1]. BC is more common in men, with a 3:1 male predominance of cases in 2019 [1]. Histologically, localized BC is subdivided into two distinct clinical entities based on staging differentiated by the presence or absence of invasion of the muscularis propria layer. This distinction is important in the risk-stratification and treatment algorithm of patients with localized BC. As with all cancers, imaging plays a significant role in the management of patients with BC, including the locoregional staging and evaluation for distant metastatic disease, which cannot be assessed at the time of cystoscopy and biopsy/resection [2]. Computed tomography (CT) is the most commonly utilized imaging modality and demonstrates excellent performance for the evaluation of nodal and distant visceral metastatic disease. Magnetic resonance imaging (MRI) has been evaluated for locoregional staging, including the evaluation of muscularis propria invasion. Positron emission tomography (PET) with [^18^F]fluorodeoxyglucose (FDG) is valuable in the whole-body staging and restaging of many cancers, and its use in cases of suspected metastatic BC or follow-up staging in cases undergoing treatment for metastatic BC is common. Other PET tracers have also been investigated for potentially improved diagnostic capability due to altered urinary excretion profiles. We aim to review these imaging modalities used in the staging and restaging of BC and the potential advantages and limitations of each imaging modality.

## 2. Computed Tomography

### 2.1. Initial Diagnosis

**Figure 1:**
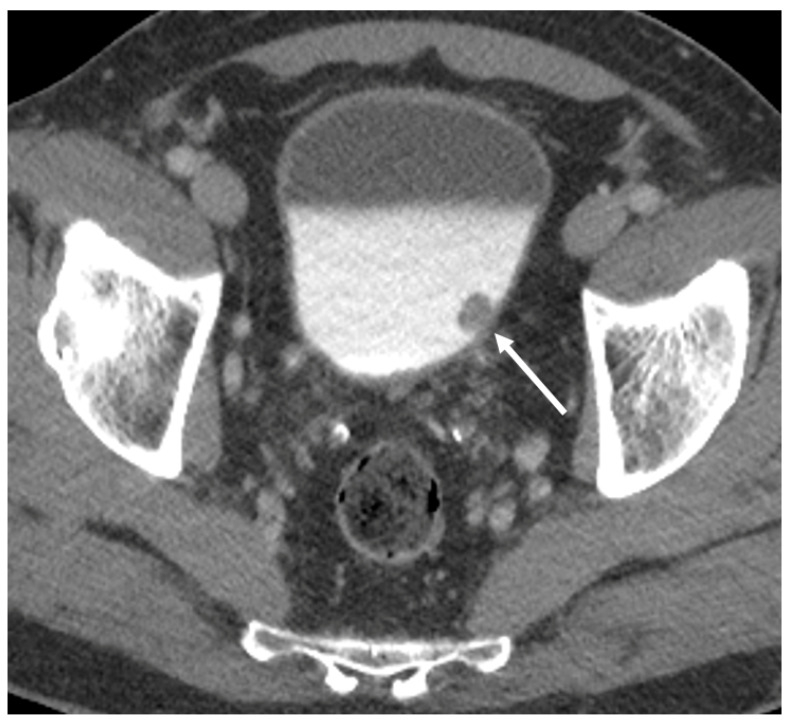
Axial CT images from the excretory phase of a CT urogram performed for hematuria demonstrating a polypoid filling defect arising near the left ureteral orifice (arrow), highly suspicious for bladder cancer.

Computed tomography (CT), specifically CT urography (CTU), is the most commonly used imaging method worldwide to diagnose and stage urothelial malignancies, for the localization, locoregional staging, and detection of distant metastases. A recent review by Mirmomen et al. demonstrated a 91% diagnostic accuracy in the detection of urothelial cancers [3]. Given the heterogeneity of bladder cancer and its variable natural history, routine screening outside of high-risk populations is not currently recommended [4]. The most common presenting symptom is gross hematuria, which is typically followed by direct visual examination via cystoscopic evaluation and imaging by means of a CTU (as it is one of the first-line modalities for gross hematuria) (Figure 1), commonly performed in tandem as the two examinations are complementary. CTU offers characterization of the upper urinary tract, with approximately 2–4% of patients with BC having concurrent upper tract urothelial carcinoma [5,6]. For the evaluation of potential BC, intravenous contrast is necessary, and thus the use of CT as an imaging modality is limited in patients with severe anaphylaxis allergies to iodinated contrast.

There are two typical approaches to how a CTU is performed. The first is with a “split-bolus” two-phase exam with a noncontrast phase and a single urothelial and excretory phase exam by administering a partial bolus, waiting until excretion, and then administering the remainder to better enhance the renal/urothelial parenchyma. The alternative is a three-phase exam consisting of noncontrast, urothelial, and delayed excretory imaging. While the “split-bolus” does save patient radiation exposure, it comes at the potential cost of masking small lesions compared with a dedicated urothelial phase alone.

A review performed by Sadow et al. demonstrated a negative predictive value of CTU of 98% and a sensitivity of 79% in patients with hematuria [7]. Some studies have shown CTU to be similarly sensitive overall to cystoscopy (CTU up to 87% sensitive, 99% specific; cystoscopy 87% sensitive, 100% specific), but it can miss very small or flat lesions that are more easily detected by cystoscopic evaluation [8]. If cystoscopy is performed first, the primary role of CTU in this setting is to detect isolated or concurrent upper tract lesions.

There are some limitations of CTU in the diagnosis of BC. Trinh et al. evaluated the reasons for false-positive and false-negative examinations when compared with cystoscopy [9]. False-positive results were from interpretation errors, most commonly due to benign prostatic hypertrophy mimicking a bladder lesion, followed by bladder trabeculation, posttreatment changes, and intravesical blood clots. False-negative results primarily resulted from flat urothelial lesions as mentioned above, though others originated due to image artifacts and inadequate bladder lumen filling during the excretory phase of the CTU imaging study. Of note, when differentiated by a contrast phase (corticomedullary, nephrographic, and excretory phases), the sensitivity and negative predictive value were highest in the corticomedullary phase (95% and 99%, respectively) [10].

Although rare (~1.5% of all bladder tumors), one area where CT exceeds cystoscopic accuracy is tumors arising within a bladder diverticulum, as diverticula may have narrow necks or otherwise may be difficult to access, limiting visualization and tissue sampling via the cystoscopic approach [11]. Tumor width and length of contact with the diverticular wall were identified as prognostic indicators and associated with stage- and cancer-specific survival.

### 2.2. Staging

While CT is suboptimal compared with MRI (discussed later) for local staging up to T3a and for differentiating non-muscle-invasive bladder cancer (NMIBC) (≤T1) from muscle-invasive bladder cancer (MIBC) (≥T2), it is quite useful for differentiating between tumors staged up to T3a from higher-staged T3b and T4 BCs [12,13]. Therefore, CT imaging is best used locally in the assessment of higher-staged larger tumors. Mirmomen et al. conducted a review of CT staging studies and demonstrated 49–93% accuracy in detecting perivesicular invasion with tumors staged ≥T3 [3]. There are some early models using machine learning that may help stratify tumors into stage <T2 and stage ≥T2, which may increase CT utility in this arena of local staging in the future, but currently, this differentiation requires further investigation and validation prior to clinical implementation and acceptance [14].

CT is useful in determining lymph node involvement in the abdomen and pelvis. Lymph node size is the predominant way nodal metastases on CT are suspected; however, abnormal morphologies such as rounded or irregular nodes may be clues to metastatic involvement. N-staging in BC is as follows: N1 cancers are a single regional node in the perivesical, obturator, internal/external iliac, or sacral lymph node chains; N2 cases have multiple regional nodes in these regions; and N3 cases have common iliac chain nodal involvement [15]. Accepted measurements for abnormal, pathologically enlarged lymph nodes are 10 mm in the abdomen and 8 mm in the pelvis as measured in their short axis [16]. Accuracy expectedly varies in multiple studies, ranging from 54 to 86%, and using lower or higher cutoffs may increase rates of false positives or negatives, respectively [3]. Notably, a size criterion of 10 mm may produce false positives in up to 30% [17]. Future tools such as machine learning and radiomic models may increase accuracy in nodal detection, but need further study [18].

Metastatic disease is generally well recognized by CT. In a review by Rajesh et al., 6% of all-comer biopsy-proven patients with BC demonstrated distant metastases, most commonly in the retroperitoneal lymph nodes, all of which were associated with cases of MIBC patients, with none in the NMIBC cohort [19]. Peritoneal metastases were described, ranging from 7.6 to 16% of cases, with increasing frequency in atypical variant histologic cases of BC [20,21].

### 2.3. Restaging

Restaging CT exams are generally considered appropriate for patients with NMIBC with symptoms or risk factors or MIBC following treatment courses as surveillance [22]. These can be performed as either standard contrast-enhanced CT scans or as CT urography. Low-risk NMIBC patients do not typically require serial surveillance CT exams or routine upper tract surveillance evaluations [22]. Risk factors for NMIBC recurrence/progression include tumors ≥3 cm, multiple tumors, carcinoma in situ, known recurrence and shortened time frame for recurrence, higher tumor grade, increased tumor stage, lymphovascular invasion, prostatic urethral invasion, variant histology, and poor response to intravesical Bacillus Calmette–Guerin (BCG) immunomodulation therapy [23]. MIBC patients have a higher risk of recurrence, with risk factors reported as advanced tumor stage, lymph node involvement, lymphovascular invasion, high-grade tumor, and positive margins. Sites of recurrence include pelvis and surgical bed recurrence, iliac or obturator node involvement, and distant, end-organ, and osseous metastases. CTU has good results in detecting recurrent bladder tumors in high-risk NMIBC cases. A study of high-risk patients demonstrated 59 bladder recurrences in 121 patients (a total of 38 positive patients) with better accuracy in the nephrographic phase imaging than in the excretory postcontrast phase imaging (91.7% vs. 83.2%) [24]. Upper tract recurrences were also better seen in the nephrographic phase imaging than in the excretory phase imaging (86.7% vs. 80%), although both are proven quite useful for diagnosis [24].

Future directions for CT may implement machine learning, neural networks, and radiomic processing protocols. Recent publications have identified that computerized decision-support systems improve radiologist accuracy and reduce interobserver variability for identifying treatment response, including complete response to neoadjuvant chemotherapy in MIBC [25,26,27].

Overall, CT is an excellent modality for initial diagnosis of bladder cancer, given its accessibility, speed, and relative cost, and is complementary to cystoscopy and urine studies, including cytopathology. It is the most commonly utilized modality for overall primary staging and restaging BC and also offers imaging evaluation to detect patients with concurrent upper tract urothelial carcinoma. Trade-offs include radiation exposure and less utility for accurate differentiation of local T-staging compared with other imaging modalities.

## 3. Ultrasound

Ultrasound is a commonly utilized imaging modality that is widely available and low-cost in relation to advanced imaging modalities such as MRI and PET/CT. Frequently, ultrasound may be the initial examination performed in patients with hematuria, as it is recommended as the initial diagnostic evaluation for microscopic hematuria by the American College of Radiology (ACR) Appropriateness Criteria [5]. Incidental bladder lesions may be detected on initial ultrasound evaluation, but little data exist regarding the use of ultrasound for the local staging of bladder cancer. A recent study evaluating the feasibility and accuracy of high-resolution microultrasound imaging for the local staging of bladder cancer found that utilizing a 29 MHz transducer, microultrasound was able to clearly delineate layers of the bladder wall and showed promising accuracy in the assessment of muscularis propria invasion [28]. However, a known limitation of ultrasound is its inability to provide comprehensive abdominopelvic staging in the setting of malignancy, and its use is likely limited to the screening of the urinary bladder and/or its possible use in local staging.

## 4. Magnetic Resonance Imaging

### 4.1. Why MRI for Bladder Cancer?

About 90% of BC are urothelial carcinoma, and approximately 75–85% of patients present with early-stage NMIBC. NMIBC is commonly diagnosed and treated with transurethral resection of bladder tumor (TURBT) and/or intravesical therapy [3,29,30]. NMIBC is considered a stage precursor to MIBC, if undetected or left untreated [31]. MIBC (≥T2 disease) may be treated with partial or radical cystectomy with pelvic lymph node dissection, neoadjuvant or adjuvant chemotherapy, and radiation. The accurate detection of MIBC is, therefore, important for prognosis and for determining a treatment strategy, which differs dramatically than for NMIBC.

Historically, TURBT has been both a form of definitive treatment for NMIBC and a local-staging strategy for NMIBC, but studies have shown that TURBT may understage 30–50% of patients [29,30,32,33]. Patients with MIBC who are understaged are at significant risk for disease progression to metastases and worse outcomes with approximately 30% higher 5-year cancer-specific mortality [29]. The suboptimal performance of TURBT for local staging, taken together with the necessity of cross-sectional imaging for nodal and metastatic staging, emphasizes the need for more advanced imaging techniques with optimization of accurate locoregional staging performance.

### 4.2. Multiparametric Bladder MRI

**Figure 2:**
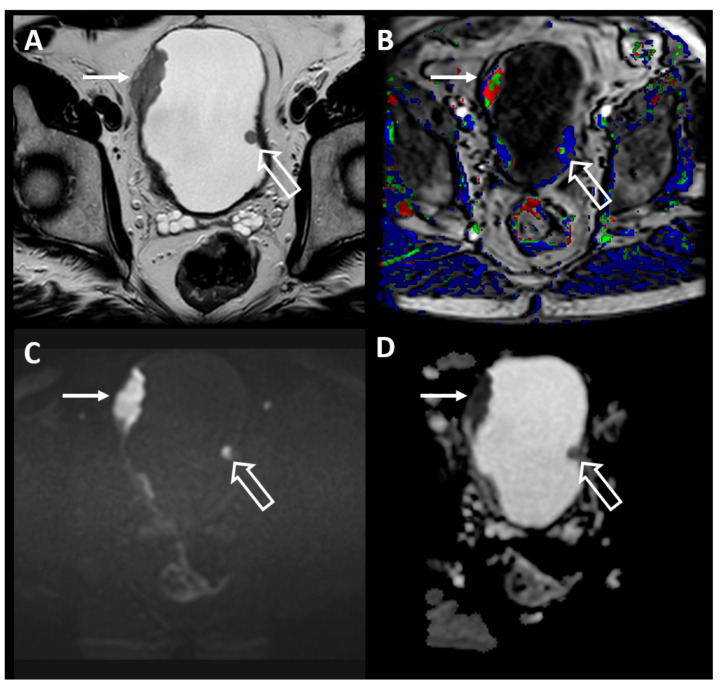
Axial T2-weighted (T2W) (**A**), dynamic-contrast enhanced (**B**), b2000 diffusion-weighted (**C**), and apparent diffusion coefficient (**D**) images of the pelvis in a patient with known multifocal bladder cancer. There are multiple intraluminal masses, one of which (closed arrow) clearly invades through the bladder wall and is consistent with muscle-invasive bladder cancer (MIBC), while the other polypoid mass (open arrow) does not disrupt the T2 hypointense bladder wall, consistent with non-muscle-invasive bladder cancer (NMIBC).

MRI is currently the superior imaging modality for soft-tissue resolution, which allows for more accurate locoregional BC staging than CT [30,34]. However, the use of MRI may be limited in some patients due to potential safety concerns with implanted metallic devices or foreign bodies. Thus, it is important to identify any potential safety hazards prior to performing an MRI for BC staging. A multiparametric MRI (mpMRI) examination consists of anatomic T2-weighted (T2W) images, functional diffusion-weighted images (DWI), and dynamic contrast-enhanced (DCE) images (Figure 2). Using mpMRI has further improved MRI for BC staging [31].

T2W anatomic images are essential for the local staging of bladder cancer, as they depict the relationship of the tumor tissue to the normal bladder wall [3]. Multiplanar fast or turbo spin-echo two-dimensional (2D) T2W images should be acquired in at least two planes (axial, sagittal, and/or coronal) with a slice thickness of 3–4 mm to maximize spatial resolution while maintaining an adequate signal-to-noise ratio. These T2W images should be acquired without fat saturation [31,35]. Similar to the recommendations for Prostate Imaging-Reporting and Data System (PI-RADS) version 2.1 used for prostate cancer detection and risk stratification, Vesical Imaging-Reporting and Data System (VI-RADS) recommends that isotropic three-dimensional (3D) T2W images serve as an adjunct [35]. The benefits of isotropic 3D sequences are that the acquisition is more rapid and the tumor base can be reformatted in any arbitrary plane. However, there is debate about the soft-tissue contrast and in-plane resolution of 3D acquisitions versus 2D T2W acquisitions in the literature, resulting in the current adjunct recommendation [36,37].

DWI captures the Brownian movement of free-water molecules, and tumors with high cellularity restrict this movement, resulting in high-signal intensity. The corresponding apparent diffusion coefficient (ADC) map is a quantitative representation of restricted diffusion with decreased signal corresponding to tumors of increased cellularity and aggressiveness [3]. Free-breathing spin-echo echo-planar imaging (EPI) DWI with spectral fat saturation should be acquired in at least two orthogonal planes (axial and sagittal and/or coronal). For accurate anatomic localization, the DWI planes and field of view (FOV) should match those used for the T2W and DCE images. An ADC map should be generated with at least two b-value sequences required; the b-values should include a high b-value of 800–1000 s/mm^2^ to achieve sufficient contrast resolution compared with surrounding tissues [31,35,37]. There are multiple strategies for accelerating the acquisition of DWI that can be employed, including parallel imaging, increasing the number of excitations, and potentially using the simultaneous multislice (SMS) technique [38].

Angiogenesis in tumors leads to hypervascularity and early contrast enhancement, which can be captured with DCE imaging. DCE images are multiple rapidly acquired T1-weighted (T1W) fat-suppressed gradient echo sequences obtained before, during, and after the intravenous (IV) administration of a low-molecular-weight extracellular gadolinium-based contrast agent (GBCA) typically in 30 s increments, although shorter time intervals can be acquired. Either 2D or 3D acquisitions can be acquired; however, 3D acquisitions are preferred for DCE imaging due to higher spatial resolution [31,35].

The T2W, DWI, and DCE sequences should include the entire bladder, proximal urethra, and pelvic lymph node chains. The prostate and seminal vesicles should be included for males, and the vagina and reproductive organs (if present) for females [31,35]. Additional T1W spin-echo larger-FOV images extending from the aortic bifurcation to the symphysis pubis should also be acquired, ideally before and after IV GBCA, to help identify hemorrhage and clot burden in the bladder lumen, enlarged pelvic lymph nodes, and bone metastasis.

### 4.3. Patient Preparation and Equipment

To minimize falsely overstaging BC by the radiologist, which may be related to reactive changes in the bladder wall, particularly after recent cystoscopic instrumentation, the MRI examination should be performed before endoscopic management or at least 2 weeks after cystoscopic procedures (TURBT or bladder biopsy) or intravesical treatment [31,35]. Similarly, if the patient’s clinical status allows, the MRI should be performed at least 2 days after removal of an indwelling catheter or instrumentation to minimize artifacts related to intravesicular gas, particularly on diffusion-weighted images (DWIs). Optimal bladder distention is essential to minimize folds or trabeculations being mistaken for BC. Conversely, an overly filled bladder can lead to increased patient discomfort during the examination and excessive motion artifact. Patients should empty their bladder 1–2 h prior and drink 500–1000 mL of water 30 min before the MRI examination. A scout image should be obtained to assess bladder distension. If further distension is needed, the patient should be encouraged to drink additional fluids, and imaging should be delayed up to 30–60 min [31,35]. Bowel motion can be minimized by the intramuscular administration of an antispasmodic agent, such as glucagon, and by employing saturation bands [30].

MRI for BC can be performed successfully on both 1.5T and 3T imaging systems; however, 3T MRI has been shown to perform better than 1.5T MRI for spatial and temporal resolution, signal-to-noise ratio, contrast-to-noise ratio, and the differentiation of cancer from normal tissues [39]. A multichannel phased array external surface coil is recommended to further optimize image acquisition [31,35].

### 4.4. MRI Findings in Bladder Cancer

There are four defined layers of the bladder wall: the urothelium, which lines the bladder lumen; the vascular lamina propria (submucosa); the muscularis propria; and the outermost serosa [30]. Three separate bladder wall layers can be seen on the imaging: the inner layer, which includes the mucosa (urothelium) and the submucosal layers (lamina propria); the muscularis propria (detrusor); and the perivesical fat. These three layers of the bladder cannot, however, be reliably differentiated on all MRI sequences [31,35].

The highly vascular inner layer (urothelium and lamina propria) is not well visualized on T2W imaging and DWI, but can be seen during DCE imaging when it enhances avidly early (approximately 20 s after GBCA administration). In distinction, the muscularis propria enhances later, slowly and progressively (approximately 60 s after GBCA administration) [40]. On T2W imaging and DWI the inner layer is not seen, and the muscularis propria is seen as a low-signal intensity line on T2W imaging and intermediate linear signal on DWI [31,35]. Finally, the inner and outer muscle fibers of the muscularis propria are oriented longitudinally, but these distinct layers are usually not apparent [30]. Nevertheless, by using all of the sequences in mpMRI, it is possible to discriminate between stage ≤T1 (tumor has not reached the muscle layer) and ≥T2 (tumor has grown into either the superficial, inner-half (T2a) or deep, outer-half (T2b) muscle layer of the bladder wall) or higher-staged tumors into or beyond perivesical adipose tissues [31]. A multi-institutional multireader study of VI-RADS demonstrated a pooled area under the curve (AUC) of 0.87 depending of the level of reader experience for differentiating MIBC from NMIBC confirmed at surgical resection [41].

Lesions suspicious for BC will have T2W imaging signal hypointense to urine, but hyperintense to muscle. Suspicious lesions will also have high signal on DWI and low signal on ADC with early postcontrast enhancement. There is a positive correlation between tumor size and grade, with larger tumors most commonly demonstrating higher-grade lesions [35]. Also, noting tumor location is important for staging, as bladder neck cancers have a higher rate of muscle invasion [35].

### 4.5. Imaging and Reporting Bladder Cancer with VI-RADS

VI-RADS is an mpMRI scoring system developed in 2018 to standardize the imaging and reporting of BC on MRI. VI-RADS incorporates tumor appearance on T2W imaging, DWI, and DCE imaging to assess risk of tumor invasion [31]. Tumors receive an overall score of VI-RADS 1–5 with VI-RADS 1 indicating an intact muscularis propria and no tumor infiltration and VI-RADS 5 indicating extension of tumor to extravesical fat. VI-RADS 1 and 2 are unlikely to invade the muscularis propria, while VI-RADS 4 and 5 are likely to infiltrate or extend beyond the bladder wall. VI-RADS 3, like PI-RADS 3, is indeterminate for muscle invasion.

The overall VI-RADS score incorporates three categories, including a structural category that is assessed on T2W imaging and includes tumor size, growth pattern, morphological features, and location. The final overall VI-RADS score is predominately based on the structural category, given the high spatial resolution of T2W imaging. There are also categories for DWI and DCE imaging, which assess the presence of muscle invasion and these categories are incorporated into the scoring of VI-RADS 3–5 lesions [35]. If there is discordance between the structural T2W imaging score and the DCE imaging score regarding muscle invasion, the findings on high-quality DWI should prevail to maximize accuracy [31].

Both retrospective and prospective studies have investigated the validity and reproducibility of VI-RADS with encouraging results. The six validation studies completed to date overall assessed approximately 1109 cases that had an average rate of 31% MIBC. Interobserver reader agreement for VI-RADS was good to excellent, ranging from 0.73 to 0.92. Sensitivity for distinguishing NMIBC from MIBC (typically set at VI-RADS 3) was between 76 and 95%, and specificity between 44 and 93% across these six studies [31].

### 4.6. TNM Staging of Bladder Cancer with MRI

MRI is the cross-sectional imaging modality of choice for assigning T-stage in patients with known BC [30,34,40,42,43]. MRI is being increasingly used for the preoperative local staging of BC because of its high soft-tissue contrast resolution with resultant anatomic detail that allows for the accurate assessment of bladder wall invasion depth [30,40]. Beyond the structural visualization afforded by T2W imaging, the functional sequences of mpMRI (e.g., DWI and DCE imaging) have further improved tumor staging performance [44]. For example, there are statistically significant improvements in specificity and accuracy in differentiating T1 and lower-grade tumors from T2 and higher-grade tumors when DWI and DCE imaging are added to T2W imaging [44].

In addition to staging tumors, MRI is able to depict perivesical fat tissue extension, regional lymph node involvement, and the presence of pelvic sidewall or osseous metastases. Second only to T-stage, nodal status is the most important prognostic variable and correlates with decreased 5-year disease-free survival [40]. Nodal metastasis correlates with tumor stage with nodal disease present in approximately 30% of T2 cancers and 60% in T3 or T4 tumors. Given this strong correlation, lymphadenectomy is usually performed in patients with MIBC even in cases of partial cystectomy [40].

BC typically spreads to the obturator nodes; however, nodal involvement can also be seen in other perivesicular or regional nodal stations, such as the hypogastric, external iliac, and presacral nodes [30,40]. Nodal disease on MRI is identified by size (>8 mm in short axis) and morphological criteria, such as round shape, irregular borders, central necrosis, and loss of fatty hilum. In TNM staging, there are four stages of lymph node (LN) involvement based on anatomic location, as opposed to the number of LNs or size criterion. N1 disease involves one regional LN, while N2 involves more than one regional LN. Involvement of an inguinal LN is N3 disease, and spread to a nonregional LN above the iliac chains is considered metastatic or M1a disease.

Given its reliance on size, MRI has limited sensitivity for the detection of nodal disease in BC by missing micrometastases and lacking specificity for distinguishing other causes of LN enlargement, such as inflammation. Incorporating DWI, particularly high b-value sequences, to improve the detection of nodal disease beyond size and morphology alone leads to marginal improvement in sensitivity and specificity [30,40]. Overall, MRI has higher specificity than sensitivity for the detection of nodal disease; therefore, if LNs are suspicious on MRI, an extended lymphadenectomy to include those nodes is warranted. Similarly, given its lower sensitivity, an absence of nodal disease on MRI should not preclude lymphadenectomy [40].

Approximately 5–15% of patients with MIBC have metastatic disease at presentation, and the most frequent sites of BC metastasis are LNs, lung, liver, and bones [40,45]. BC also has a high rate of local and distant metastatic disease; for example, in patients who undergo cystectomy, the rate of distant metastasis has been reported to be 70%, and local 30% [40]. In patients with MIBC, CT examination of the chest, abdomen, and pelvis with contrast remains the imaging modality of choice for the assessment of distant metastases. However, acceleration techniques in MR together with more advanced MR imaging techniques, for example, PET/MR and whole-body MRI, hint at a paradigm shift to MR for full TNM staging of BC in the not-too-distant future.

## 5. Molecular Imaging (PET/CT and PET/MRI)

### 5.1. Why PET/CT for Bladder Cancer?

PET/CT is a commonly utilized imaging modality for patients with a wide variety of cancers in both initial staging and restaging. The most commonly utilized radiotracer is [^18^F]fluorodeoxyglucose (FDG), which is a radiolabeled glucose analog that is taken up by the GLUT2 transporter that is overexpressed in many cancer cells. Typically, FDG-PET/CT is performed for the whole-body staging of cancers. The radiotracer is injected approximately 60 min prior to the scan, and the patients are instructed to follow a specific diet and activity regimen prior to the exam to avoid muscle activity and alterations in radiotracer biodistribution due to endogenous insulin release. A review of the Surveillance, Epidemiology, and End Results (SEER) database from 2004 to 2011 demonstrated that a total of 36,855 patients underwent a PET/CT scan for bladder cancer of any stage and that the number of scans markedly increased throughout the time period, resulting in excess national spending of $11.6 million for this imaging modality [46,47]. However, for patients with bladder cancer, FDG-PET/CT has the potential to change patient management, and patients with positive FDG-PET/CT scans demonstrate worse overall survival and higher recurrence rates in cases of recurrent disease [48].

### 5.2. FDG-PET/CT for Bladder Cancer Staging

**Figure 3:**
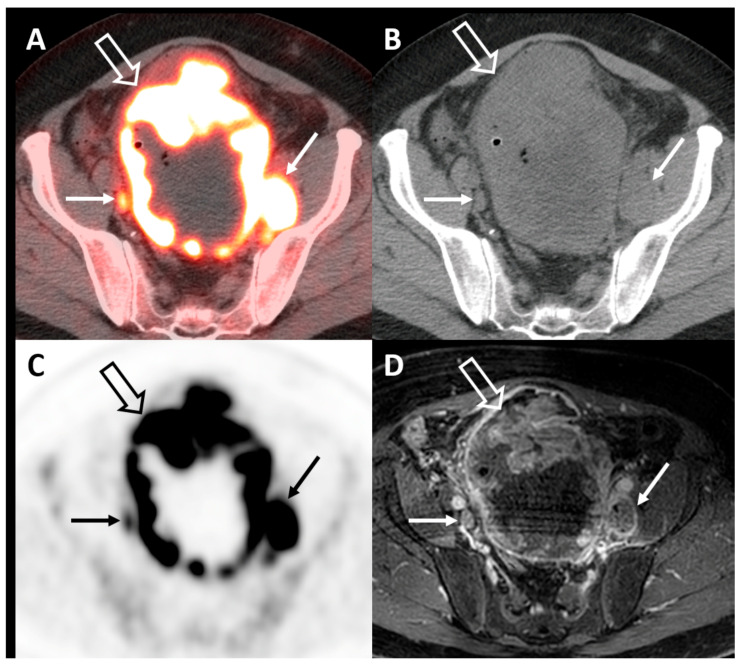
Axial fused FDG-PET/CT (**A**), CT (**B**), PET (**C**), and corresponding T1-fat saturated postcontrast MRI (**D**) demonstrating an extensive hypermetabolic mass involving nearly all of the bladder wall (open arrow) and multiple pelvic lymph node metastases (closed arrow), some of which measure less than 1 cm in short axis and are more easily identified on PET/CT vs. CT or MRI alone.

FDG-PET/CT has been extensively studied in the setting of the initial staging of bladder cancer. Most often, this is applied to the setting of MIBC where the risk of pelvic lymph node metastases is far greater than that for NMIBC and useful for preoperative risk stratification (Figure 3). The current ACR Appropriateness Criteria for the pretreatment staging of MIBC rate FDG-PET/CT as “may be appropriate” [49]. Traditionally, FDG-PET/CT for bladder cancer has been limited by a number of factors, including false-positive findings due to inflammation and obscuration of the urinary bladder by radiotracer activity in excreted urine. Different techniques for improving the imaging of the urinary bladder on FDG-PET/CT exist, including the administration of diuretics and delayed-time-point imaging [50,51]. Several studies have evaluated the diagnostic accuracy of FDG-PET/CT in patients with bladder cancer. In a combined meta-analysis and single-institution study of patients with bladder cancer scheduled to undergo radical cystectomy and pelvic lymph node dissection, the authors determined that the pooled sensitivity and specificity for detection of lymph node metastases were 57% and 95%, respectively [52]. These findings were also confirmed in two subsequent meta-analyses, which demonstrated a pooled sensitivity of 56–57% and a pooled specificity of 92% for the detection of lymph node metastases on FDG-PET/CT [53,54]. A small (*n* = 15) study evaluating the use of FDG-PET/CT for the preoperative lymph node staging of MIBC demonstrated minimal benefit of FDG-PET/CT compared with CT alone, thought to potentially be due to overlap in standardized uptake values (SUVs) between inflammatory and malignant lesions [55]. A strength of FDG-PET/CT and molecular imaging is the detection of metastatic disease in lymph nodes that are not pathologically enlarged by size criterion. A study evaluating FDG-PET/CT in patients with bladder cancer undergoing radical cystectomy found that using a short-axis diameter of 10 mm for lymph node enlargement, FDG-PET/CT outperformed CT, but when the threshold for lymph node enlargement was decreased to 8 mm, no significant difference in accuracy was observed [16]. An integrated approach for pelvic lymph node staging in patients with bladder cancer prior to radical cystectomy that included both maximum SUVs and lymph node size criterion improved the accuracy of FDG-PET/CT [56]. As immunotherapy aimed at PD-1/PD-L1 inhibition becomes increasingly utilized in the treatment algorithm for bladder cancer, there is interest in non-invasive methods of evaluating PD-1/PD-L1 expression. A study evaluating the association between findings from FDG-PET/CT and those from PD-1/PD-L1 testing found that higher FDG uptake by bladder cancer was associated with elevated PD-1/PD-L1 expression [57].

### 5.3. FDG-PET/CT for Neoadjuvant Chemotherapy Response and Restaging

Current National Comprehensive Cancer Network (NCCN) guidelines recommend that patients with MIBC undergo neoadjuvant chemotherapy prior to evaluation for radical cystectomy. As with many cancers, the evaluation of response to neoadjuvant chemotherapy is of utmost importance, as this may lead the patient to undergo surgery or become surgically ineligible. While changes in lesion size can be used to track response to therapy, the metabolic information gained from FDG-PET/CT can also provide important information. Frequently at the time of diagnosis, patients with bladder cancer undergo transurethral resection of their tumor. Immediately following this and at follow-up, it can be difficult to assess for residual/recurrent tumor on cross-sectional imaging depending on the amount of tumor that was initially resected. A study evaluating delayed-time-point FDG-PET/CT performed within 1 month of initial transurethral resection in 79 patients found that residual tumors at subsequent confirmatory biopsy had higher mean and maximum SUV and greater lesional thickness when compared with inflammatory changes [51]. Following neoadjuvant chemotherapy in patients with MIBC, FDG-PET/CT demonstrated 75% sensitivity and 90% specificity in identifying patients with complete pathological response at cystectomy [58]. FDG-PET/CT is also accurate in distinguishing primary tumor downstaging from nonresponse in bladder cancer, which can have important implications in potential alterations of ineffective neoadjuvant chemotherapy regimens [59]. Following treatment, FDG-PET/CT has been shown to be highly valuable in the detection of recurrent disease in patients with bladder cancer. In a study of 41 patients with bladder cancer who underwent FDG-PET/CT for restaging, recurrent biopsy-proven bladder cancer was confirmed in 20 of 21 (95.2%) patients who had a positive FDG-PET/CT scan and resulted in changes in treatment decisions in approximately 40% of the patients [60]. Additionally, in this same study a negative PET/CT study was associated with longer progression-free survival and overall survival [60]. A study evaluating the accuracy of FDG-PET/CT compared with conventional imaging with contrast-enhanced CT/MRI and chest X-ray found that for the detection of recurrent bladder cancer, the sensitivity and specificity of PET/CT were 94% and 76%, respectively, compared with 87% and 55% for conventional imaging [61]. A recent meta-analysis demonstrated a pooled sensitivity of 94% and a pooled specificity of 92% for FDG-PET/CT in the detection of recurrent or residual bladder cancer [62].

### 5.4. Alternative PET Tracers in Molecular Imaging of Bladder Cancer

While FDG remains the most commonly utilized radiotracer in oncologic imaging, its pitfalls have led to research on other radiotracers. The two most studied alterative radiotracers used in the imaging of bladder cancer are [^11^C]choline and [^11^C]acetate. Choline is a naturally occurring small molecule incorporated into tumor cells via phosphorylation by choline kinase. Acetate is a naturally occurring metabolic substrate that enters the fatty acid metabolic pathway. Both of these pathways/processes are upregulated in a number of cancers. However, a limitation of these radiotracers is the short half-life of ^11^C (20 min), which requires an on-site cyclotron for production and significantly limits geographic distribution. A systematic review and meta-analysis of the diagnostic accuracy of [^11^C]choline and [^11^C]acetate for lymph node staging in patients with bladder cancer found a pooled sensitivity of 66% and a pooled specificity of 89% [63].

### 5.5. PET/MRI in Bladder Cancer

PET/MRI scanners are being increasingly installed and utilized throughout the world, leading to increased interest in appropriate applications of these scanners in clinical use. In particular, pelvic imaging is well suited for PET/MRI owing to the superior soft-tissue characterization gained by PET/MRI compared with PET/CT. For bladder cancer, the information gained from both PET and MRI is complementary and can lead to increased confidence in reporting findings, increased sensitivity of detection, and overall improvement in comprehensive staging in a single imaging examination. Given the novelty of this new hybrid imaging modality, there is limited research in the field of bladder cancer with PET/MRI. A pilot study of 21 patients with MIBC who underwent preoperative FDG-PET/MRI found that FDG-PET/MRI had a performance similar to that of CT for the detection of the primary tumor, but the detection of lymph node metastases were limited by the lower number of patients with true pathological lymph node involvement [64]. A separate prospective pilot study of patients with bladder cancer who underwent FDG-PET/MRI found that the additional PET information helped to appropriately characterize the level of suspicion for findings in the bladder, pelvic lymph nodes, and remainder of the pelvis when compared with MRI alone [65]. Of note, the PET portion of the exam increased suspicion of findings in 52% of the patients (36% increase and 64% decrease), and 95% of the changes were deemed correct [65]. An additional pilot study evaluating acetate-PET/MRI in 18 patients with bladder cancer found low sensitivity for the detection of lymph node metastases, but promising data for response to neoadjuvant chemotherapy [66]. Given the small size of these pilot studies, larger studies are needed to adequately evaluate and confirm the potential applications and value of PET/MRI for bladder cancer.

## 6. Conclusions

Bladder cancer imaging through multiple modalities provides important information in the staging and restaging of patients with BC. CT is the most widely available and frequently utilized imaging modality for BC and demonstrates good performance for the detection of nodal and visceral metastatic disease. MRI offers potential value for the locoregional staging and evaluation of muscular invasion of BC, which is critically important for prognostication and treatment decision-making. FDG-PET/CT is somewhat limited in the evaluation of the primary BC due to urinary excretion of the radiotracer, but shows good performance for the detection of nodal and visceral metastatic disease and offers the potential to detect metastatic disease in lymph nodes that are not anatomically enlarged. FDG-PET/MRI is a novel hybrid imaging modality with potential advantages of both MRI and FDG-PET/CT in a single-setting comprehensive staging examination, but scanner availability and limited research on the topic limit its widespread use.
